# The (Neutrophils + Monocyte)/Lymphocyte Ratio Is an Independent Prognostic Factor for Progression-Free Survival in Newly Diagnosed Multiple Myeloma Patients Treated With BCD Regimen

**DOI:** 10.3389/fonc.2020.01617

**Published:** 2020-09-02

**Authors:** Yanbin Pang, Hong Shao, Ziheng Yang, Lixia Fan, Wenwen Liu, Jianhong Shi, Yuqing Wang, Ying Han, Lin Yang

**Affiliations:** ^1^Department of Hematology, Affiliated Hospital of Hebei University, Baoding, China; ^2^Department of Hematology-Oncology, General Hospital of Shenzhen University, Shenzhen, China; ^3^Department of Hematology, Second Hospital of Hebei Medical University, Shijiazhuang, China; ^4^Medical College, Medical Department of Hebei University, Baoding, China; ^5^Central Laboratory, Affiliated Hospital of Hebei University, Baoding, China; ^6^Department of Cell Morphology, Affiliated Hospital of Hebei University, Baoding, China

**Keywords:** multiple myeloma, (neutrophils + monocytes)/lymphocytes ratio, prognostic factor, progression-free survival, bortezomib

## Abstract

**Objective:**

Bortezomib is one of the important drugs that have made breakthrough progress in multiple myeloma (MM) in the past 10 years. However, the heterogeneity of its efficacy makes it difficult to predict the risk of disease progression. The purpose of this study is to determine the prognostic significance of the (neutrophils + monocytes)/lymphocytes ratio (NMLR) in newly diagnosed MM patients who received BCD regimen therapy in terms of progression-free survival (PFS).

**Methods:**

A total of 150 patients who fulfilled the International Myeloma Working Group (IMWG) criteria were enrolled in the study retrospectively. The prognostic value of NMLR was evaluated by 150 patients with MM who were treated with BCD (bortezomib + cyclophosphamide + dexamethasone) regimen therapy. NMLR was calculated by the ratio of (neutrophils + monocyte) to lymphocytes. According to receiver operating characteristic curves, the cutoff value was 1.90. The patients were divided into high NMLR group (H-NMLR, NMLR ≥1.90) and low NMLR group (L-NMLR, NMLR <1.90). The clinical characteristics, treatment responses and PFS of the two groups were analyzed.

**Results:**

The median age of the patients was 61 years. Fifty-five (36.67%) patients showed lower NMLR at initial diagnosis. Although NMLR was unable to discriminate prognosis in ISS stage I/II patients, interestingly, the addition of NMLR to the ISS further defined prognosis particularly in stage III. Low-NMLR group who achieved early immune reconstruction significantly higher than that of the high-NMLR group (*P* < 0.001). NMLR value was 1.98 ± 1.02 for the patients who achieved early immune reconstruction, which was 3.26 ± 2.52 for the patients without immune reconstruction (*P* < 0.05). Compared with the H-NMLR group, the levels of β2-microglobulin, serum creatinine and calcium were lower, and the very good partial response or better (≥VGPR) ratio was higher in L-NMLR group. The L-NMLR group experienced a superior median PFS compared with the H-NMLR group (24.0 versus 15.5 months; *P* < 0.001). In addition, several other prognostic factors of PFS were estimated, including the high-risk cytogenetics, β2-microglobulin and the depth of treatment response 3 months after treatment with BCD regimen. Moreover, NMLR was an independent predictor of PFS including non-high risk cytogenetics (0.587; *P* = 0.031).

**Conclusion:**

In patients with newly diagnosed MM undergoing BCD regimen, the NMLR <1.90 was an independent prognostic factor for PFS as well as early immune reconstruction and lower disease burden.

## Introduction

The overall survival of multiple myeloma (MM) patients has greatly increased in recent years. Bortezomib is one of the important drugs that have made breakthrough progress in MM in the last decade. However, MM remains an incurable disease (1–3). In the condition of no transplantation, most of the patients died of disease progression within 2 years (4). One of the researches focuses on MM is to explore the factors of disease progression and to adjust the treatment regimen to prolong the progression-free survival (PFS) of patients (5). The early lactate dehydrogenase level and the international prognosis score systems, cannot accurately predict the MM response during new drug treatment. Therefore, molecular and genetic abnormalities in plasma cells have been gradually applied in MM’s prognostic staging system to predict patients’ response to new drugs in recent years (5, 6). However, it is still largely unknown how to accurately predict the patient’s response to new drugs, which may be due to the factors only reflect the biological characteristics of clonal plasma cells and ignore the role of immune factors in the disease progression. More and more studies showed that multiple myeloma is a kind of tumor disease with both abnormal plasma cells and immune system, in which immune failure is the important factor for disease progress (7–9). In addition, more and more evidence indicated that the key to improve the anti-tumor effects of anti-tumor drugs is to further eliminate the residual tumor cells by the anti-tumor immunological reaction. Moreover, the success of CAR-T cells in MM treatment also showed that the recovery of patient’s immune function plays an important role in further improving the efficacy of treatment of MM (10–12). Therefore, the immunosuppressive state of MM patients at diagnosis may be an important reason to limit further improvement of the efficacy (13, 14). How to evaluate the overall immune status of patients is an urgent problem in clinical practice. Recent researches reported that neutrophils inhibit the activation of T cells by reactive oxygen species, and monocytes as precursors of tumor-associated macrophages are closely related to the formation of MM immunosuppressive microenvironment, and absolute lymphocyte counts are independent prognostic factors for survival (14–16). Although there are some controversies, some evidence demonstrated that absolute neutrophils count (ANC) to the absolute lymphocyte count (ALC) ratio (NLR) and ALC to the absolute monocyte count (AMC) ratio (LMR), as immune state indicators of MM patients, play important roles in disease progression (14, 17, 18), which indicated that both neutrophils and monocytes are indispensable components in the complex immune microenvironment of MM and play different roles (19). Therefore, the ratio of (neutrophils + monocytes)/lymphocytes (NMLR) may be an effective surrogate parameter for reflecting the immune status of MM patients. This study analyzed the effect of NMLR on the PFS of patients with MM who received a combination of BCD regimen (bortezomib, cyclophosphamide, dexamethasone) to explore the effect of immune status on patient response to treatment, and provided a theoretical basis for the application of immunotherapy in MM.

## Materials and Methods

### Patients and Methods

We identified all patients with newly diagnosed MM at Affiliated Hospital of Hebei University between 01/2016 and 10/2019 by retrospective chart review. Patients were treated with BCD regimen (bortezomib 1.3 mg/m^2^ d1, 4, 8, 11; dexamethasone 20 mg d1-2, 4-5, 8-9, 11-12, cyclophosphamide 300 mg d1, 4, 8), which was repeated every 28 days. Patients who achieved partial response or better after 6–8 cycles of BCD regimen were treated with the original regimen every 3 months for 2 years, and if the disease progressed or unacceptable toxicity, patients switch to other treatment regimes. The study was approved by the Ethics Committee of Affiliated Hospital of Hebei University. All patients were diagnosed according to the IMWG diagnostic criteria (20). Patients were excluded from the study, including: diabetes mellitus, coronary or congestive heart failure, chronic pulmonary disease, hepatocirrhosis, infectious events and for solid tumor. Data collected including age, gender, bone marrow examinations and complete blood counts as well as calcium, creatinine, albumin, β2-microglobulin, free light chain, and immunoglobulin concentrations which were obtained at the time of diagnosis. Complete blood counts were collected 1 month after BCD treatment. Fluorescence *in situ* hybridization (FISH) was performed on diagnostic bone marrow specimens and high-risk cytogenetics were defined as del(17p), t(14;16), t(4;14)and t(14;20). Definitions of response and progression were used according to the consensus criteria of International Myeloma Working Group (21). Very good partial response (VGPR) or better (≥VGPR) = VGPR + near CR (near complete response, nCR) + CR (complete response, CR). PFS was defined as the time from diagnosis to disease progression, any event, death as a result of any causes, or last follow-up, whichever occurred first. The NMLR value was calculated using data obtained from the complete blood count as follows: NMLR = (neutrophil count + monocyte count)/lymphocyte count.

### Statistical Analyses

The main objective of the study was to determine the influence of NMLR at the time of MM diagnosis on PFS. Secondary endpoints were to evaluate the influence of NMLR on VGPR rate at 3 months. The optimal cut-off value for the NMLR was determined to be predicted for PFS by receiver operating characteristic curves (ROC) and area under the curve (AUC). The correlation of NMLR with various parameters was assessed with Pearson’s chi-square test (or Fisher’s exact test) for categorical parameters and with student *t*-test for continuous parameters. We compared baseline characteristics and clinical outcomes between two groups that were dichotomized according to the NMLR cutoff value. Kaplan–Meier analysis was conducted to estimate the survival of patients, and the differences between survival curves were tested for statistical significance using the two-tailed log-rank test. Multivariate Cox regression analyses were performed to adjust for potential confounders. Variables with a *P* value of less than 0.1 on univariate analysis were entered into the multivariate model. Statistical differences with a *p* value <0.05 were considered significant. All analyses were performed using SPSS statistical software (version 19.0).

## Results

There were 150 patients with newly diagnosed MM who met the inclusion criteria. Baseline characteristics of patients were listed in Table 1. The median age was 61 years (range 35–81 years); 93 patients (62.0%) were men and 57 patients (38.0%) were women. Overall, 60 patients (40.0%) had IgG, 42 (28.0%) had IgA, 13 (8.7%) had IgD and 35 (23.3%) had light chain disease. Eighty-one patients (54.0%) presented with International Staging System stage III myeloma. Cytogenetic data were available for 135 of the 150 patients. Forty-five (33.33%) patients were diagnosed with high risk FISH/karyotype. Forty-three (28.67%) patients presented with renal insufficiency. Treatment response was ≥VGPR in 58 (38.67%) patients. Death prior to progression occurred in only eight patients (5.3%) in the entire cohort. The median follow-up duration was 16.4 (range: 0.7–35.8) months.

**TABLE 1:** Baseline patient characteristics based on the absolute NMLR ratio.

**Characteristic**	**All cases (%)**	**L-NMLR (%)**	**H-NMLR (%)**	***P* value**
Patients	150	55(36.67%)	68(63.33%)	
Age, years, median (range)	61(35−81)	62(41−79)	61(35−81)	0.755
Age, years ≥60 years	94(62.67%)	36(65.45%)	58(61.05%)	0.591
Males, *N* (%)	84(56.0%)	57(60.0%)	27(49.09%)	0.195
**M-fraction type (%)**				0.979
IgG	60(40.88%)	23(41.82%)	37(38.95%)	
IgA	42(37.32%)	15(27.27%)	27(28.42%)	
IgD	13(8.67%)	5(9.09%)	8(8.42%)	
Light chain	35(23.33%)	12(21.82%)	23(24.21%)	
**ISS**				
Stage I	24(16.00%)	10(18.18%)	14(14.74%)	0.141
Stage II	45(30.00%)	21(38.18%)	24(25.26%)	
Stage III	81(54.00%)	24(43.64%)	57(60.00%)	
NMLR (median, range)	2.50(0.32−12.33)	1.50(0.32−1.89)	3.17(1.91−12.33)	
Hemoglobin (g/L, median, range)	83.50(41.00−161.00)	78.00(50.00−127.00)	86.00(41.00−161.00)	0.061
LDH	167.00(63.00−723.00)	145.00(69.00−502.00)	167.00(63.00−723.00)	0.516
Albumin (g/L)	33.8(17.40−51.25)	33.20(19.80−51.25)	33.95(17.40−49.40)	0.311
β2-microglobulin (mg/L)	5.80(1.36−88.40)	4.80(1.80−23.30)	6.26(1.36−88.40)	0.003
Serum creatinine	87.50(32.00−743.00)	76.00(38.70−520.0)	107.0(32.00−743.00)	0.000
Calcium	2.28(1.30−3.90)	2.22(1.61−3.53)	2.30(1.30−4.90)	0.047
Plasma cell percentage, (%)	31.50(10−98.00)	27.00(10.50−98.00)	32.50(10−92.00)	0.290
≥VGPR ratio (%)	58(38.67%)	27(49.09%)	31(32.63%)	0.046
Abnormal karyotype	45(33.33%)	18(36.00%)	27(31.76%)	0.614

*Abbreviations: LDH: lactate dehydrogenase; ISS, international staging system; VGPR, very good partial response; NMLR, (neutrophil + monocytes) to lymphocytes ratio.*

### Baseline Patient Characteristics According to NMLR at Diagnosis of MM

**FIGURE 1:**
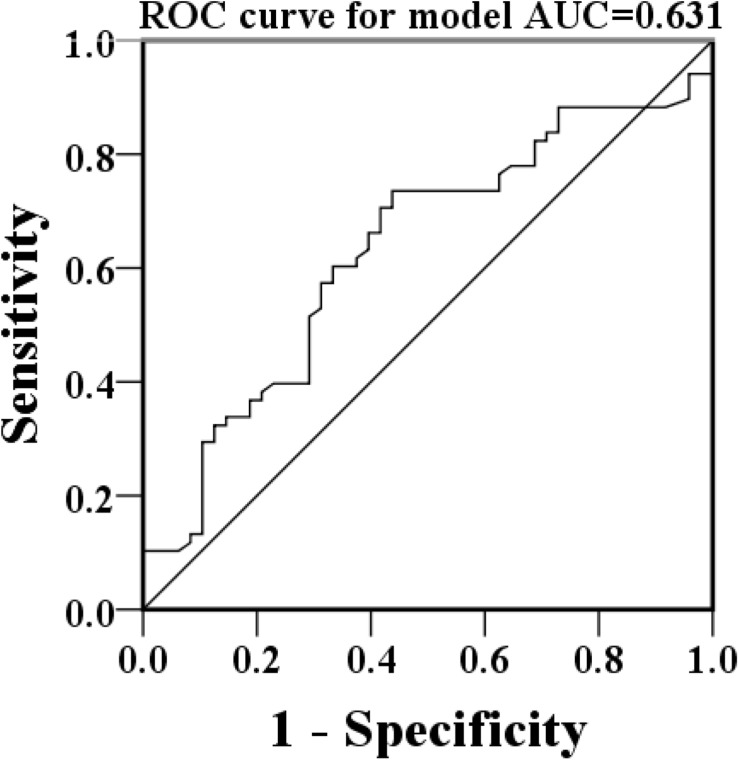
The cutoff values provided by ROC analysis for NMLR for the prediction of in MM patients. The area under the ROC curve for NMLR was 0.631, *P* = 0.016. Abbreviations: ROC, receiver operating characteristic; AUC, area under the curve. NMLR, (neutrophil + monocytes) to lymphocytes ratio.

The median NMLR was 2.50 (range: 0.32–12.33). We performed ROC curve analysis to calculate optimal cut-off points for the NMLR. The optimum cut point was the point at which the sensitivity and specificity were highest for the PFS. The optimum cutoff point was 1.90 (with a sensitivity of 73.5% and a specificity of 56.2%), with an AUC value of 0.631 [95% confidence interval (CI): 0.528–0.734, Figure 1]. Based on the cutoff points for the NMLR, patients were separated into two groups: high-NMLR group (H-NMLR, NMLR ≥1.90, *n* = 95) and low-NMLR group (L-NMLR, NMLR <1.90, *n* = 55). L-NMLR group had lower β2-microglobulin, serum creatinine, and calcium levels and had higher ≥VGPR percentage than those with H-NMLR group. In our study, there was no significant difference between the two groups with respect to age, albumin, hemoglobin and ISS stage, plasma cell percentage or cytogenetics (Table 1).

### Univariate Analyses

**FIGURE 2:**
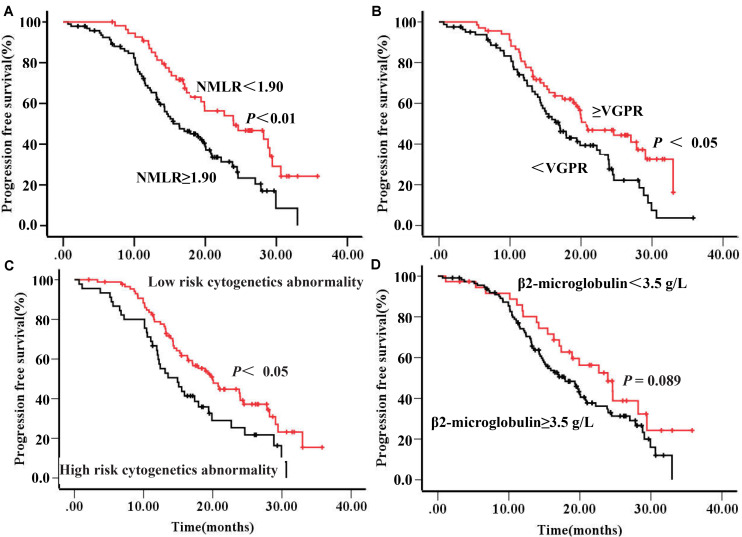
Results of analysis of parameters as predictors of relapse-free survival in patients with newly diagnosed multiple myeloma. **(A)** Progress free survival by the NMLR at diagnosis. **(B)** Progress free survival by therapeutic response. **(C)** Progress free survival by karyotype. **(D)** Progress free survival by theβ2-microglobulin. Abbreviations: VGPR, very good partial response. NMLR, (neutrophil + monocytes) to lymphocytes ratio.

At a median follow-up time of 16.4 months, the median PFS of the entire cohort was 19.4 months. Kaplan-Meier analysis was performed to determine if the NMLR were associated with PFS. The patients with NMLR <1.90 at diagnosis had better PFS when compared to those with a NMLR ≥1.90 (24.40 and 15.50 months, respectively, *P* = 0.002) (Table 2 and Figure 2A). These results suggested that NMLR levels at diagnosis were able to discriminate MM patients receiving BCD regimes with regard to PFS. Additional covariates that were significantly associated with PFS in our cohort were treatment response (≥VGPR versus <VGPR) 3 months after treatment with BCD regimen and cytogenetics abnormality category (High-risk versus Low-risk) at diagnosis (Table 2 and Figures 2B,C, respectively). There was a trend toward improved PFS with lower β2-microglobulin level (23.93 versus 17.90 months, *P* = 0.089) (Table 2 and Figure 2D).

**TABLE 2:** Univariate analysis for progression-free survival.

**Factors**	**Univariate analysis**	
	**Median PFS (95% CI)**	***P* value**
Age ≥60 years	18.90 (15.12–22.68)	0.885
Age <60 years	19.70 (16.24–23.16)	
Hemoglobin ≤100 g/L	17.90 (14.03–21.50)	0.481
Hemoglobin >100 g/L	22.67 (16.33–29.01)	
Serum creatinine ≥177 μmmol/L	19.87 (12.83–26.91)	0.271
Serum creatinine <177 μmmol/L	19.40 (16.28–22.52)	
Albumin <35 g/L	19.70 (15.86–23.55)	0.841
Albumin ≥35 g/L	19.30 (16.30–22.30)	
β2-microglobulin <3.5 g/L	23.93 (17.39–30.47)	0.089
β2-microglobulin ≥3.5 g/L	17.90 (14.03–21.77)	
≥VGPR	20.77 (15.66–25.88)	0.012
<VGPR	16.90 (13.95–19.85)	
ISS Stage I	24.60 (16.07–33.13)	0.232
ISS Stage II	17.90 (13.71–22.09)	
ISS Stage III	19.40 (15.03–23.78)	
LDH (<240 IU/L)	19.70 (16.48–22.92)	0.572
LDH (≥240 IU/L)	17.37 (10.08–24.66)	
High risk cytogenetics	14.90 (10.93–18.87)	0.011
Non- high risk cytogenetics	19.97 (16.82–23.12)	
NMLR <1.90	24.00 (15.44–32.50)	0.002
NMLR ≥1.90	15.50 (11.57–19.43)	

*Abbreviations: LDH: lactate dehydrogenase; VGPR, very good partial response; ISS, international staging system; NMLR, (neutrophil + monocytes) to lymphocytes ratio.*

**FIGURE 3:**
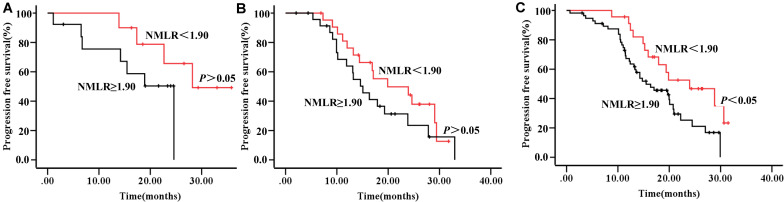
Progression-free survival **(A–C)** by ISS stages I, II, and III with NMLR < 1.90 (red line) and NLR ≥ 1.90 (black line). Abbreviations: ISS, international staging system, NMLR, (neutrophil + monocytes) to lymphocytes ratio.

In contrast to the significance of NMLR classification, ISS alone had a weak prognostic meaning in our cohorts based on PFS. We also tested if the NMLR could improve the prognostic impact of the ISS. Although NMLR was unable to discriminate prognosis in ISS stage I/II patients (Figures 3A,B, respectively), interestingly, the addition of NMLR to the ISS further defined prognosis particularly in stage III (Figure 3C), which could possibly improve the predictive value of the ISS staging system and assist in stage III individualizing therapies.

### Multivariate Analysis

Multivariate analysis including NMLR, cytogenetics, β2-microglobulin and treatment response (≥VGPR versus <VGPR) showed that NMLR (HR = 0.404, 95% CI: 0.245–0.664, *P*-value <0.001) as well as treatment response (HR = 0.587, 95% CI: 0.361–0.953, *P*-value <0.05) were independent predictors of PFS. There was a trend toward improved PFS with therapeutic response (HR = 0.631, 95% CI: 0.393–1.014, *P*-value = 0.057) (Table 3).

**TABLE 3:** Multivariate analysis for progression-free survival.

**Factors**	**Multivariate analysis**	***P* value**
	**HR (95% CI)**	
NMLR <1.90	0.404 (0.245–0.664)	0.000
Non-high risk cytogenetics	0.587 (0.361–0.953)	0.031
β2-microglobulin <3.5 g/L	0.941 (0.544–1.629)	0.829
≥VGPR	0.631 (0.393–1.014)	0.057

*Abbreviations: NMLR, (neutrophil + monocytes) to lymphocytes ratio; VGPR, very good partial response.*

### The Effect of NMLR on Early Immune Reconstitution in MM

**FIGURE 4:**
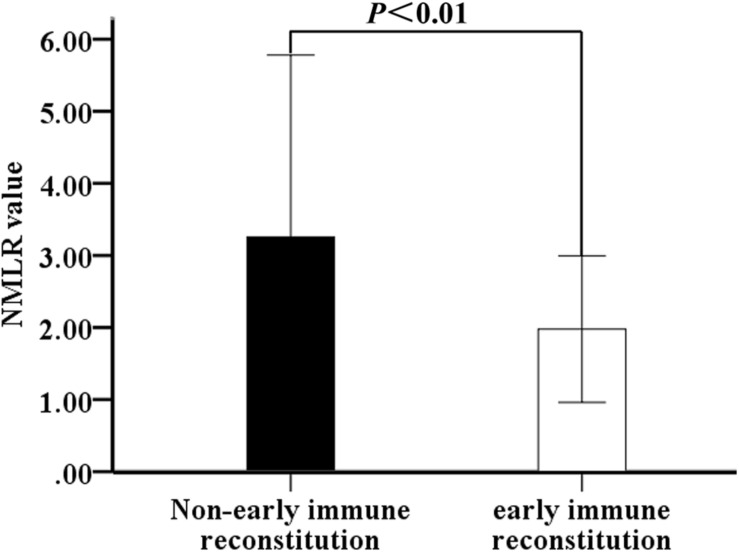
NMLR in newly diagnosed MM patients based on early immune reconstruction. Abbreviation: NMLR, (neutrophil + monocytes) to lymphocytes ratio.

The early immune reconstitution of multiple myeloma is defined as the recovery of absolute lymphocyte count and monocyte counts to normal reference range 1 month after treatment initiation (22). Therefore, we analyzed the effect of NMLR on early immune reconstitution in MM patients. The reference range for absolute lymphocyte count and absolute monocyte count was 0.8–4.0 × 10^9^/L and 0.1–0.6 × 10^9^/L, respectively, in our institution. Excluding the influence of infection and chemotherapy, the ALC and AMC in peripheral blood of 121 patients were analyzed 1 month after BCD treatment. Twenty-three (32.86%) of 70 patients with H-NMLR achieved early immune reconstruction significantly lower than that with L-NMLR (60.78%, *P* < 0.001, Table 4). Moreover, the value of NMLR was 1.98 ± 1.02 for the patients who achieved early immune reconstruction, which was 3.26 ± 2.52 for the patients without being achieved immune reconstruction (*P* < 0.01, Figure 4). These results indicated that high NMLR is a negatively factor for early immune reconstitution in MM patients.

**TABLE 4:** The impact of NMLR at diagnosis on for the effect of early immune reconstitution.

**Factors**	**NMLR <1.90**	**NMLR ≥1.90**	***P* value**
Early immune reconstitution	31 (60.78%)	23 (32.86%)	0.002
Non-early immune reconstitution	20 (39.22%)	47 (7.14%)	

*Abbreviation: NMLR, (neutrophil + monocytes) to lymphocytes ratio.*

## Discussion

Although the outcome have been significantly improved during the last 20 years by the proteasome inhibitors and immunomodulators, MM is still an incurable disease in most patients due to primary or acquired drug resistance (14). It remains a challenge prediction of prognosis in MM, especially in the era of novel agent (6). ISS staging system is commonly used as a prognostic tool which is based on tumor burden and thus neglects markers of immune dysfunction (17). In recent years, with the improving understanding of immune mechanism in the pathogenesis of MM, it has gradually transformed into the progress of clinical treatment. The use of monoclonal antibodies and CAR-T cells in clinical treatment has significant improvement of relapsed/refractory MM patients, indicating that immune abnormalities play important roles in the pathogenesis of MM (12). However, how to evaluate the real *in vivo* balance of immune system is still an urgent problem.

Peripheral blood absolute lymphocyte count (ALC), absolute neutrophil count (ANC) and absolute monocyte count (AMC) have been used as good surrogate markers of immune surveillance in several solid tumors and hematologic malignancies including MM (23–26). In MM, ANC/ALC ratio (NLR) and ALC/AMC ratio (LMR) have been found to be independent prognostic factors at the time of diagnosis, before, and after autologous hematopoietic stem cell transplantation (13, 17–19, 22). Most of them concluded that elevated NLR and low LMR could predict poor prognosis. However, the research results were not completely consistent, Romano et al. (17) results showed that patients with higher NLR had shorter PFS than patients with lower NLR However, Wang et al. (18) results indicated that NLR had no significant effect on PFS in newly diagnosed MM patients. In addition to the heterogeneity of patients and different treatment regimen, it was also an important reason that the NLR itself cannot fully reflect the suppressive effect of immune suppression factors on the immune system. For example, Romano et al. (19) showed monocyte may have a different biological role in the complex network of the bone marrow immunologic microenvironment which supports myeloma growth. In addition, the results of Lee et al. (13) showed that the immunosuppressive factors represented by dNLR which was calculated by the formula of dNLR = ANC/(WBC – ANC) were the poor prognosis factors of PFS and OS, the formula seems to ignore the inhibition of monocyte on the immune system. Theoretically, the immune balance in patients *in vivo* can be better reflected by neutrophil plus monocyte/lymphocyte ratio. Therefore, we incorporated both LMR and NLR into one score, (neutrophil + monocyte)/lymphocytes ratio (NMLR), to predict the progression in patients with MM treated with BCD therapy.

Here we demonstrated the importance of NMLR at the time of diagnosis in determining risk of progression in patients with MM treated with BCD. Our results demonstrated that patients with L-NMLR had a higher ≥ VGPR ratio and a longer duration of PFS, especially in patients with ISS Stage III. These results were consistent with several other studies, which showed that immunosuppressive microenvironment is a poor prognostic factor for MM disease progression (8, 13, 14, 17, 18). There are several explains for these results. Firstly, the peripheral blood ANC and AMC are precursor cell for myeloid-derived suppressor cells (MDSC) and tumor-associated macrophages (TAM), respectively, which release growth and survival factors promoting tumor evasion of the host defense mechanisms (14, 17). On the contrary, infiltrating lymphocytes were associated with favorable prognosis, as was recently shown in patients with MM (16, 27). Therefore, NMLR will be a good surrogate marker of the real *in vivo* balance of immune system. The higher NMLR means that the immune system is severely inhibited, which plays a critical role in the development of MM from its precursor condition, monoclonal gammopathy of undetermined significance (MGUS), in part by allowing immune tumor evasion (7). On the other hand, apoptotic cancer cells elicited by chemotherapy induce T cells to enter the local microenvironment of tumor by releasing chemokines, which can further improve the chemotherapeutic effect (10). Therefore, immunosuppressive microenvironment reduces the sensitivity of tumor cells to chemotherapy. We proposed that patients with severely suppressed immune system at diagnosis may be candidates for approaches targeting T cell exhaustion such as immune checkpoint blockade or other novel therapies. Our hypothesis was indirectly supported by Guillerey et al. (9) results that T cell immunoglobulin and ITIM domains (TIGIT), an immune checkpoint receptor known to negatively regulate T cell function, can be blocked by monoclonal antibodies, which increases the effecter function of MM patient CD8 + T cells and suppresses development in mice.

Another important finding of this study was that the proportion of patients with high NMLR who achieved early immune reconstitution after 1 month of treatment with BCD was significantly smaller than that of patients with low NMLR, and the NMLR value at diagnosis of patients who achieved early immune reconstitution was significantly lower than those without early immune reconstitution. These results suggested that the treatment based on bortezomib may not overcome the effect of immunosuppressive microenvironment on the poor prognosis of MM, which may be related to the mechanism of bortezomib (14, 22). The possible explanation for this finding is the specific mechanism of action of bortezomib. Bortezomib inhibits inducible NF-κB activity in MM cells, but unexpectedly enhances constitutive NF-κB activity via activation of the canonical pathway, which elevate sheparanase protein expression and tumor necrosis factor alpha (TNF-α) production (28). TNF-α is known to promote PD-L1expression in cancer cells, which contributes to immune escape of tumor cells (29). In addition, it has been shown that bortezomib inhibits the expression of co-stimulatory molecules and depletes alloreactive T lymphocytes (30). Binder and Dosani’s (22, 26) results also demonstrated that bortezomib could not effectively restore the immune function of MM. Therefore, bortezomib combined with immunomodulators or treatment methods to restores patients’ immune function may further improve the treatment effect of patients. The searches for immune biomarkers that help stratify patients based on their immune status is important for patients to choose new drugs. As this may help to determine which patients are most likely to benefit from the upcoming immunotherapy.

The disadvantage of this study is that as a retrospective study, the NMLR need to be further validated in independent prospective studies. Despite such limitation, our results and other research results clearly showed that the prognosis of patients is not only related to the genetic abnormalities of plasma cells but also the immune status at diagnosis (17–19, 22). Novel therapeutic regimens will need to consider the effect of genetic determinants as well as immune status in future clinical trials.

In summary, our results suggested NMLR easily measurable predictors of PFS in treatment-naive MM patients treated upfront with BCD; MM patients with high NMLR were not easily to achieve the recovery of early immune reconstruction as well as higher disease burden. This information could be integrated with ISS staging to personalize the treatment. Ongoing and future studies will need to elucidate the underlying mechanisms and answer the question if the negative prognostic impact of overall immunosuppression can be overcome by therapeutically induced in patients with multiple myeloma.

## Data Availability Statement

All datasets generated for this study are included in the article/supplementary material.

## Ethics Statement

The study was approved by the Ethics Committee of the Affiliated Hospital of Hebei University.

## Author Contributions

LY and YH designed the study. YP, HS, and ZY were involved in data acquisition. LF and JS performed the statistical analysis. YP edited the manuscript. YW and JS corrected and reviewed the manuscript. All authors discussed and interpreted the results and gave their final approval to the definitive manuscript.

## Conflict of Interest

The authors declare that the research was conducted in the absence of any commercial or financial relationships that could be construed as a potential conflict of interest.
